# Coccolithophore community response along a natural CO_2_ gradient off Methana (SW Saronikos Gulf, Greece, NE Mediterranean)

**DOI:** 10.1371/journal.pone.0200012

**Published:** 2018-07-02

**Authors:** Maria V. Triantaphyllou, Karl-Heinz Baumann, Boris-Theofanis Karatsolis, Margarita D. Dimiza, Stella Psarra, Elisavet Skampa, Pierros Patoucheas, Nele M. Vollmar, Olga Koukousioura, Anna Katsigera, Evangelia Krasakopoulou, Paraskevi Nomikou

**Affiliations:** 1 National and Kapodistrian University of Athens, Faculty of Geology and Geoenvironment, Panepistimioupolis, Athens, Greece; 2 University of Bremen, Geosciences Department, Bremen, Germany; 3 Hellenic Centre for Marine Research, Institute of Oceanography, Anavyssos, Attiki, Greece; 4 Aristotle University of Thessaloniki, Department of Geology, Thessaloníki, Greece; 5 University of the Aegean, Department of Marine Sciences, Lesvos, Greece; University of Connecticut, UNITED STATES

## Abstract

A natural pH gradient caused by marine CO_2_ seeps off the Methana peninsula (Saronikos Gulf, eastern Peloponnese peninsula) was used as a natural laboratory to assess potential effects of ocean acidification on coccolithophores. Coccolithophore communities were therefore investigated in plankton samples collected during September 2011, September 2016 and March 2017. The recorded cell concentrations were up to ~50 x10^3^ cells/l, with a high Shannon index of up to 2.8, along a pH gradient from 7.61 to 8.18, with values being occasionally <7. Numerous holococcolithophore species represented 60–90% of the surface water assemblages in most samples during September samplings. *Emiliania huxleyi* was present only in low relative abundances in September samples, but it dominated in March assemblages. Neither malformed nor corroded coccolithophores were documented. Changes in the community structure can possibly be related to increased temperatures, while the overall trend associates low pH values with high cell densities. Our preliminary results indicate that in long-termed acidified, warm and stratified conditions, the study of the total coccolithophore assemblage may prove useful to recognize the intercommunity variability, which favors the increment of lightly calcified species such as holococcolithophores.

## Introduction

The cumulative emissions in anthropogenic CO_2_ from 1870 to 2014 totaled about 545 GtC; almost half of these emissions remain in the atmosphere and increase the potential to enhance climate change [1]. In addition, the oceans absorb approximately 30% of the atmospheric CO_2_ produced by anthropogenic activities [1–4]. As a result, the concentration of bicarbonate ions is increasing; causing simultaneous reduction in carbonate ions, decline of ocean pH and lowering of the calcium carbonate saturation state (Ω) of both calcite and aragonite [e.g., 1, 2, 5, 6]. During the last 200 years, surface ocean pH has fallen almost 0.1 units to a current day global average of approximately 8.2 [7]. The associated ocean acidification with surface pH predicted to fall by up to 0.77 units till 2250 [e.g., 8, 9] comprises a major threat for marine ecosystems, particularly for marine calcifiers and consequently for the global biogeochemical cycles [5, 10–12]. Up to now, several studies investigated the acidification effects on both benthic [5, 13] and planktonic marine organisms [5, 14–21], however few of them have dealt with *in situ* field data [22–25].

The semi-enclosed Mediterranean Sea is a small-scale ocean with high environmental variability and steep physicochemical gradients, all increasing towards the east [26, 27]. Particularly, the eastern Mediterranean basin lies in a climatological transition zone under the influence of both tropical and mid-latitude climate processes [28], making it highly sensitive to global climate change. Future climate scenarios predict a temperature increase larger than the global average value, reduced precipitation and increase of the interannual variability [1].

Both acidification and warming are expected to affect marine ecosystems of the Mediterranean Sea, mostly by altering microbial nutrient cycling, carbon fixation, primary production rates and therefore plankton community structure [29, 30], with documented consequences on biodiversity [31]. Interestingly, the Mediterranean offers the unique opportunity to study gradients of long-term acidification at marine volcanic CO_2_ vents lacking toxic sulphur compounds that are abundant especially around Italy and Greece. The first *in situ* field data results from Ischia site in the Tyrrhenian Sea [23, 32] revealed a dramatic shift in benthic community composition along a pH gradient with a collapse in species diversity and loss of functional groups as CO_2_ levels increase. Additional sites are now being used to test observations initially made at Ischia; e.g. Vulcano in Italy [25, 33–36] and Methana in Greece [37, 38]. CO_2_ seeps have also been shown to be useful for studying the effects of ocean acidification on plankton organisms, although CO_2_ levels may vary spatially and temporally around the seeps.

Coccolithophores (planktonic photoautotrophic protists) are currently the dominant calcifying organisms in the Mediterranean waters [39–41], an environment supersaturated with respect to calcite and aragonite [42]. They produce minute calcium carbonate plates called coccoliths, which are arranged around the individual cells forming the coccospheres. As it has been shown from a number of culture studies, coccolithophores have complex life cycles involving alternation between a haploid holococcolith-producing Mg-rich phase and a diploid heterococcolith-producing phase [43, 44]. The species composition in the Aegean Sea (NE Mediterranean) is relatively diverse and dominated mainly by the species *Emiliania huxleyi* [45], which is featured by more heavily calcified coccoliths during the cold winter-spring season [46]. High numbers of holococcolithophore cell densities seem to be the main feature of late spring-early autumn coccolithophore assemblages in the thermally stratified Aegean surface layers [45]. Scattered field studies so far [25], have shown coccolithophores to decrease significantly with decreasing pH; also species diversity progressively weakened as CO_2_ levels increased and Ω_calcite_ was lowered. Furthermore, malformed and corroded *E*. *huxleyi* coccoliths were related to low pH waters [25].

The present study aims to investigate the state and composition of coccolithophore communities under naturally acidified conditions. Main goals are to document potential effects on the assemblages along a natural CO_2_ gradient off Methana marine volcanic vent field, in order to investigate how coccolithophores respond to increased CO_2_ levels in oligotrophic areas and to assess whether responses to ocean acidification were modulated by seasonality. This may be of broader interest as nutrient-poor regions are expected to expand worldwide due to increased thermal stratification of ocean waters caused by ongoing climate change.

## Study area

**Fig 1:**
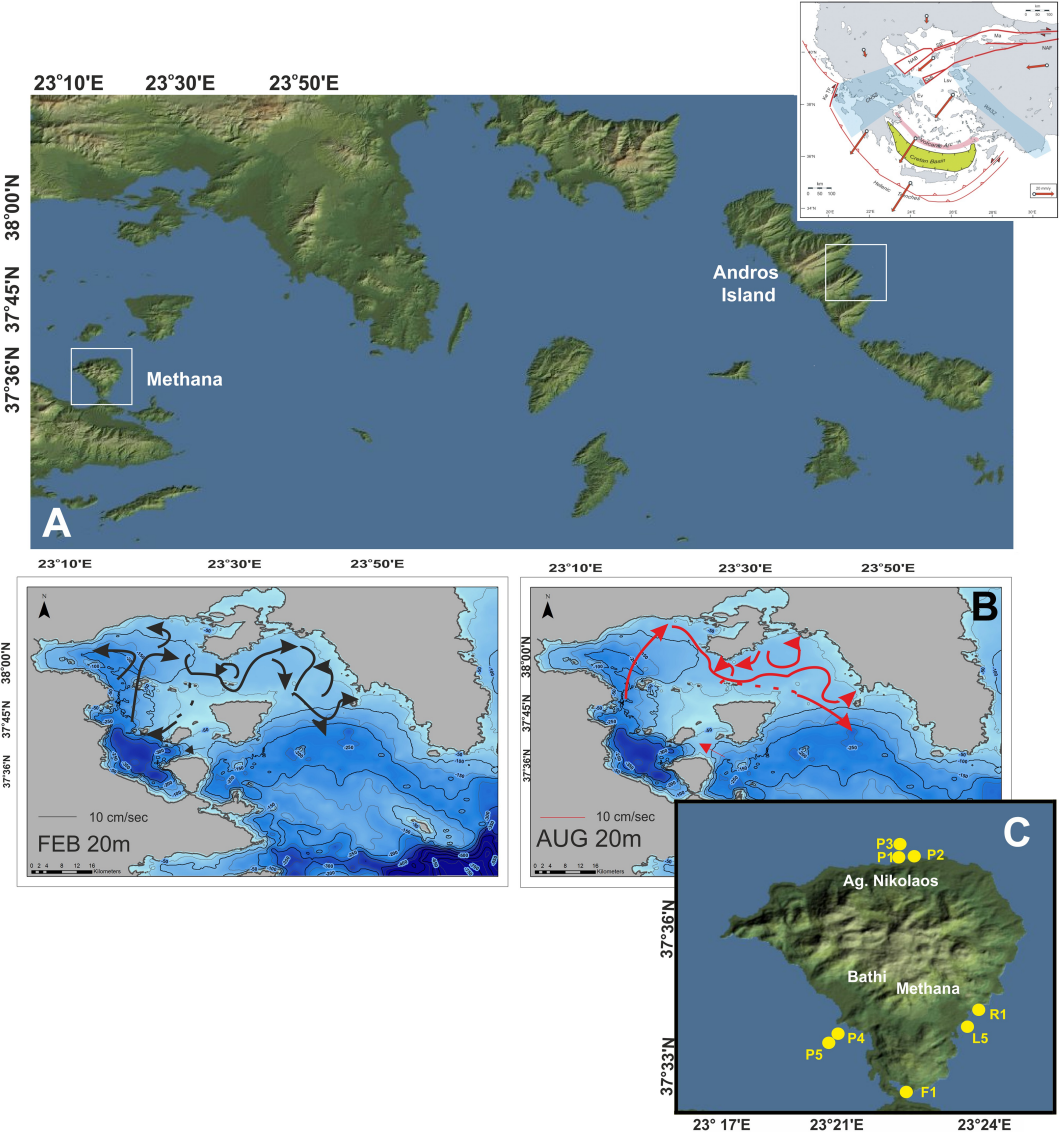
Study area. A. Map of the central Aegean Sea (NE Mediterranean) with sampling sites visited in the present study; Methana represents the western end of the Aegean Volcanic Arc. Image resource: NASA Worldview. The inlet map presents the dominant tectonic structure of the Aegean Sea domain [48]. B. Bathymetry and hydrography of Saronikos Gulf. Bathymetry data are provided by HCMR (Hellenic Centre for Marine Research). The map was designed with ArcGiS software (ESRI) v.10.4. Hydrographic data are redrawn from [46]. C. Sample location around Methana peninsula. Image resource: NASA Worldview.

The Saronikos Gulf covers an area of approximately 2600 km^2^ of complex bathymetry and geometry (Fig 1). The outer gulf, at the SE, is connected to the Aegean Sea and has depths gradually decreasing towards the inner gulf and the Attica coast from about 200 m to 100 m. The western gulf displays great depth variability, with depths locally exceeding 400 m and water masses being exchanged through the passages between Aegina and Salamina islands to the north and Aegina Island and Methana peninsula to the south (Fig 1A). Saronikos Gulf is characterized by robust seasonal flows that are induced by thermohaline effects and density contrasts with inflowing Aegean waters, and can be modified by the wind [47]. In summer an anticyclonic and a cyclonic flow exists throughout the gulf above and below the pycnocline, whereas in winter and early spring an anticyclonic flow prevails in the upper ~100 m (Fig 1B). The predominant northerly winds in summer and winter push the inner gulf eastward seasonal jet to the south, whereas northwesterly, westerly, and southerly winds favor the northward meandering of the seasonal jet in the inner gulf [47].

The volcanic area of Methana is located at the eastern Peloponnese peninsula within the southwestern area of inner Saronikos Gulf and represents the western end of the Aegean Volcanic Arc (Fig 1A [48]). The last eruption on Methana was in 230 BC as described by the ancient Greek geographer Pausanias; an active submarine volcano NW of Methana peninsula has been discovered lately [49]. On Methana peninsula there are thermal springs and mofettes and the coastal area at the northern part is still hydrothermally active with gas emissions of mainly carbon dioxide and smaller amounts of nitrogen, carbon monoxide and methane [37, 50]. The seawater chemistry together with the seasonal variability of macroalgal communities at CO_2_ seeps off Methana have already been monitored from 2011 to 2013 [37], showing that seawater pH decreased to levels predicted for the end of this century at the seep site with no confounding gradients in Total Alkalinity, salinity, temperature or wave exposure. Free sulphide concentrations were below the measurable limit (1 μM) [37]. In contrast, the samples near Loutra thermal baths (south eastern part of the peninsula) had a concentration of free sulphides of 35 μM [37].

## Materials and methods

44 water samples were collected with a single Hydrobios oceanographic bottle from 8 coastal stations off Methana peninsula during September 2011, September 2016, and March 2017, (Fig 1C and Table 1). Sampling permission was issued by the Municipality of Troizina-Methana. All samplings were conducted under mild weather conditions, i.e. with no prevailing winds. Stations P1, P2 and P3 represent the main area featured by low pH conditions due to CO_2_ emissions; P4 and P5 are pristine stations, whereas F1 was situated within an enclosed embayment hosting a small fish aquaculture plant. Stations L5 and R1 are affected by thermal springs (releasing sulphides and radium, respectively).

**Table 1:** Sample information. Stations’ location, sampling dates, physicochemical parameters, total coccolithophore density (cells l^-1^) resulting from both inverted microscopy and Scanning Electron Microscopy techniques and coccolithophore diversity (*H’*) based on SEM countings.

station	latitude (°N) longitude (°E)	date	water depth (m)	Temperature (°C)	Salinity (psu)	Chl-*a* (μg l^-1^)	inverted microscope countings (cells l^-1^)	SEM countings (10^3^ cells l^-1^)	Shannon Wiener index (*H’*)
P1	37°38'17.91"	9/2016	0	26.1	37.2	no data	no data	no data	
	23°21'36.18"	3/2017	0	15.4	38.1	0.288	200	6.87	0.84
		9/2016	2	27.1	37.1	0.21	1680	39.22	2.19
		3/2017	2	15.2	38.8	no data	no data	6.46	0.91
		9/2011	5			no data	no data	24.41	2.04
		9/2016	5	26.5	38.2	0.164	1280	24.26	1.80
		3/2017	5	15.3	39.1	0.81	360	10.06	0.80
P2	37°38'18.29"	9/2016	0	26.3	38.4	no data	no data	no data	
	23°22'2.80"	3/2017	0	15.3	38.3	no data	2600	4.54	1.13
		9/2011	5			no data	no data	5.01	1.31
		9/2016	2	27.3	38.3	0.182	1240	24.85	2.53
		3/2017	2	15.1	38.5	0.469	4356	6.58	1.15
		9/2016	5	26.5	37.9	0.177	15.903	22.52	2.24
		3/2017	5	15.5	38.6	no data	2760	5.30	1.24
P3	37°38'28.17"	9/2016	0	26.6	38.5	no data	no data	no data	
	23°21'30.71"	3/2017	0	15.5	38.5	no data	no data	1.99	0.75
		9/2016	2	27.5	38.2	0.112	1280	18.18	1.47
		3/2017	2	14.7	38.5	0.575	no data	2.38	1.11
		9/2016	10	26.5	38.7	0.183	2600	29.55	2.31
		3/2017	10	14.7	38.6	no data	no data	3.66	0.94
		9/2016	20	27.3	38.7	0.128	720	31.48	1.71
		3/2017	20	14.6	38.4	0.961	1000	2.25	0.97
		9/2016	40	24.6	38.6	0.271	1680	35.42	2.71
		3/2017	40	14.7	38.2	1.303	720	3.85	0.77
		9/2016	60	22.7	38.8	0.043	no data	19.39	1.67
		3/2017	60	14.8	38.8	0.598	1680	1.67	0.16
P4	37°34'41.26"	9/2016	0	26.2	38.5	no data	no data	no data	
	23°20'51.39"	3/2017	0	15.3	38.6	no data	no data	no data	
		9/2016	2	27.2	37.8	0.03	1440	20.15	2.28
		3/2017	2	14.7	38.2	0.328	3520	8.76	0.71
		9/2016	10	27.1	38.2	0.018	2680	14.20	2.10
		3/2017	10	14.4	38.3	no data	no data	9.65	1.06
		9/2016	20	26.3	38.3	0.025	no data	11.45	2.48
		3/2017	20	14.5	38.6	0.558	1080	7.76	1.12
		9/2016	40	25.5	38.7	0.039	no data	25.56	2.84
		3/2017	40	14.4	38.6	1.367	4560	8.02	0.56
		9/2016	60	21.2	37.9	0.118	1160	22.96	2.29
		3/2017	60	14.6	38.6	2.340	2600	7.29	0.41
P5	37°34'39.69"	9/2016	0	26.2	38.5	no data	no data	no data	
	23°20'45.11"	3/2017	2	15.1	38.4	no data	2320	no data	
		9/2016	2	26.7	38.9	no data	880	31.48	2.01
		3/2017	10	14.6	38.6	no data	5880	4,38	0.68
		9/2016	5	26.3	38	no data	880	30.43	2.44
		3/2017	20	14.5	38.2	no data	2600	no data	
R1	37°35'13.96"	9/2016	2	27.2	38	no data	2400	17.90	2.20
	23°23'54.37"	3/2017	2	17	38.3	no data	no data	no data	
		9/2016	5	27.3	38.2	no data	2840	19.15	2.07
		3/2017	5	16.3	38.5	no data	no data	5.8	0.92
F1	37°34'47.92"	9/2016	2	27.4	38.6	no data	5920	53.58	2.73
	23°23'36.69"	3/2017	2	16.7	37.5	no data	1760	no data	
		9/2016	5	26.8	38	no data	27960	no data	
		3/2017	5	15.7	38.5	no data	1760	9.24	1.12
L5	37°3'26.30"	9/2016	3	27.2	37.9	no data	1800	21.09	2.16
	23°22'12.30"								

Temperature, salinity and pH were measured using a multiprobe (YSI 63). The probe was calibrated before use with pH 4.01, 7.01 and 10.01 NBS standards; the uncertainty in using the NBS scale for seawater pH measurements (approximately 0.05) was considered acceptable [37]. Mineral nutrients were measured according to Strickland and Parsons [51] and Rimmelin and Moutin [52]. The amount of chlorophyll-*a* that corresponded to the 0.2–2.0 μm and >2.0 μm size classes was measured fluorometrically [53].

The calcium carbonate saturation state (Ω) of both calcite and aragonite were calculated with CO2Sys program configured for Excel by Pierrot et al. [54] using the current pH, temperature, salinity, phosphate and silicate measurements and the total alkalinity (AT) values resulted from monitoring of the site in 2011–2013 [37]. The set of carbonic acid apparent dissociation constants (K1 and K2) [55], the equilibrium constant of hydrogen fluoride [56], the stability constant of the hydrogen sulfate ion [57] and the boron to chlorinity ratio [58] were chosen.

For total phytoplankton analysis, 25 ml of seawater per sample were examined by inverted microscope [59, 60]. Cell density was calculated as cells l^-1^.

For coccolithophore analysis, in each sampling station 2 liters of seawater were filtered on Whatman cellulose nitrate filters (47 mm diameter, 0.45μm pore size). Salt was removed by washing the filters with about 2 ml of mineral water. The filters were dried open and stored in plastic Petri dishes.

Out of a total of 44 samples, 33 samples have been analyzed using a Zeiss DSM 940A Scanning Electron Microscope (SEM) at the University of Bremen, Department of Geosciences. A small piece of each filter (~1 cm^2^) was cut out, fixed on double-sided adhesive carbon tape to an aluminum stub and sputter coated with Au/Pd. The analysis of the filters was performed at 10 kV and more than 100 coccospheres were counted when possible at 3000x magnification. Coccolithophore cell densities were calculated as follows: Number of coccospheres l^-1^ = FxC/AxV, with F = filtration area (mm^2^), C = number of counted coccospheres, A = counted area (mm^2^) and V = filtered volume (l).

Eleven samples have been examined in a Jeol JSM 6360 SEM (National and Kapodistrian University of Athens, Faculty of Geology and Geoenvironment). A piece of each filter approximately 8x8 mm^2^ was attached to a copper electron microscope stub using a double sided adhesive tape and coated with Au. All the individual coccospheres occurring on the examined filter area were identified and counted. The absolute abundances of coccolithophore densities (cells l^-1^) were calculated following Jordan & Winter [61], by scaling up the raw counts from a known scanned area. Identification of coccolithophore species generally followed the taxonomic guides of Young et al. [62] and Malinverno et al. [63].While processing the data, all samples have been grouped in two depth classes (0–20 m and 40–60 m). Shannon Wiener diversity index (*H’*) was calculated using Past.exe 1.23 software [64] for the different depth classes in each sampling station.

## Results

### Temperature, salinity, pH, carbonate saturation state, nutrients and chl-*a*

Water temperatures during September 2016 sampling displayed relatively high values, between 22.7° C, in 60 m, and 27.5° C, in 2 m, whereas in March 2017 temperatures mostly varied within a smaller range (14.4–15.5; Table 1). Salinity was generally > 38 psu (range 37.1–38.9 psu), with lower values observed mostly in September sampling (Table 1).

Median pH values varied between 7.61 and 8.18 during September samplings, whereas even lower values (<7) have been recorded for the seep area (2011: 6.53, 2016: 6.93, station P1; Table 2) that are associated with undersaturated conditions in both calcite and aragonite [37]. In March sampling, median values varied between 7.17 and 7.92 (Table 2). It has been shown [37] that the pH variability in the area off Methana is mainly attributed to changes of the CO_2_ vent emissions and other factors (e.g. hydrogen sulphide) that would affect both pH and AT are practically missing. The additions of CO_2_ gas alter the carbonate system equilibria leaving AT constant. Assuming that AT remains relatively constant in the area (stations P1-P5) and using our pH, salinity, temperature and nutrients data, we did a rough estimation of the saturation state of both carbonate minerals with CO2Sys for the 2016–17 sampling, which shows Ω<1 in station P1 where pH minimum values have been recorded (Table 2). Nutrient and chl-*a* concentrations for all analyzed samples showed the typical oligotrophic summer Aegean Sea conditions, whereas in March the content of NO_2_ +NO_3_ reflects the seasonal nutrient enrichment and the consequent increase in chl-*a* (Table 2).

**Table 2:** Seawater carbonate chemistry, average nutrient concentrations and total chl-a values in the sampling sites.

station	SEEP (P1)			P2			P3		P3		P4		P4		P5		F1		R1		L5
depth interval (m)	0–20			0–20			0–20		40–60		0–20		40–60		0–20		0–20		0–20		0–20
sampling period	Sept-2011 (n = 40)	Sept-2016 (n = 3)	March 2017 (n = 3)	Sept-2011 (n = 26)	Sept-2016 (n = 3)	March2017 (n = 3)	Sept-2016 (n = 4)	March2017 (n = 4)	Sept-2016 (n = 2)	March2017 (n = 2)	Sept-2016 (n = 4)	March2017 (n = 4)	Sept-2016 (n = 2)	March2017 (n = 2)	Sept-2016 (n = 3)	March2017 (n = 3)	Sept-2016 (n = 2)	March2017 (n = 2)	Sept-2016 (n = 3)	March2017 (n = 3)	Sept-2016 (n = 1)
**pH min**	6.53*	6.93	7.61	7.27*	7.57	7.65	7.66	7.73	7.81	7.66	8.15	7.83	8.09	7.82	8.15	7.86	7.95	7.20	8.06	7.08	7.80
**Ω**_**Ar**_ **min**	0.09*	0.51	1.03	0.57*	1.40	1.12	1.72	1.31	2.10	1.13	4.39	1.60	3.57	1.57	4.34	1.71					
**Ω**_**ca**_ **min**	0.13*	0.34	1.60	0.88*	2.11	1.74	2.59	2.03	3.19	1.75	6.59	2.49	5.42	2.44	6.54	2.65					
**pH median**	7.69*	7.61	7.69	7.88*	7.66	7.70	7.73	7.73	7.84	7.71	8.17	7.85	8.10	7.84	8.18	7.92	7.99	7.39	8.13	7.17	7.80
**Ω**_**Ar**_ **median**	1.16*	1.51	1.23	2.3*	1.69	1.25	1.99	1.31	2.23	1.25	4.54	1.67	3.63	1.64	4.57	1.93					
**Ω**_**ca**_ **median**	2.45*	2.27	1.91	3.5*	2.54	1.94	2.99	2.03	3.39	1.95	6.83	2.60	5.52	2.54	6.88	3.00					
**pH max**	7.99*	7.61	7.77	8.13*	7.66	7.70	7.76	7.77	7.87	7.75	8.21	8.02	8.11	7.85	8.22	7.95	8.03	7.57	8.14	7.60	7.80
**Ω**_**Ar**_ **max**	3.45*	1.51	1.46	4.05*	1.69	1.25	2.12	1.42	2.37	1.36	4.86	2.36	3.70	1.67	4.89	2.05					
**Ω**_**ca**_ **max**	5.20*	2.27	2.26	6.10*	2.54	1.94	3.18	2.21	3.60	2.12	7.30	3.67	5.62	2.59	7.36	3.19					
**NO**_**3**_ **+ NO**_**2**_	0.12*	0.02	15.22	0.14*	0.03	15.32	0.04	15.49	0.08	15.71	0.04	16.38	0.05	16.21		16.40	0.07	16.02	0.24	16.27	
**NH**_**4**_	0.23*	0.14	0.42	0.26*	0.06	0.29	0.18	1.72	0.07	0.92	0.07	1.33	0.06	1.43		2.00	0.10	0.56	0.14	0.34	
**PO**_**4**_	0.025*	0.00	0.10	0.03*	0.02	0.08	0.02	0.06	0.00	0.16	0.03	0.03	0.03	0.57		0.08	0.09	0.11	0.06	0.03	
**SiO**_**2**_	4.02*^+^	0.54	0.87	6.37*^+^	0.74	0.35	0.54	0.79	0.75	2.52	0.73	0.19	0.72	0.57		0.22	0.38	0.65	2.15	0.61	
**total chl-*a***		0.374		no data	0.358	0.980	0.423	1.536	0.314	1.902	0.073	0.886	0.157	3.708							

* data from Baggini et al. (2014)

+ values referring to SiO4

### Total phytoplankton and coccolithophores

**Fig 2:**
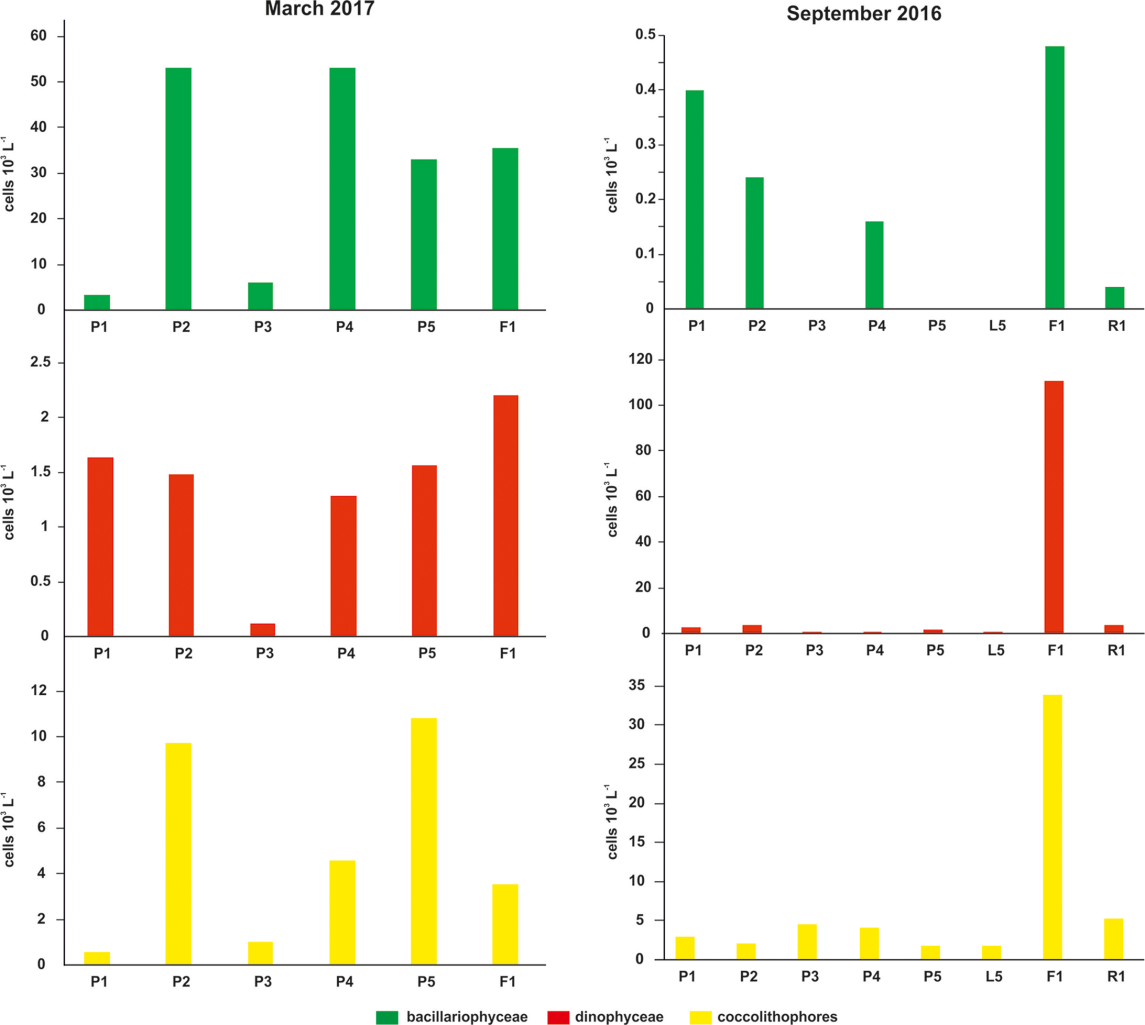
The structure of plankton community. Abundance (cells l^-1^) of the major plankton groups Dinophyceae, Bacillariophyceae and the Coccolithophores component, during the two sampling periods.

Inverted microscope total phytoplankton identifications (Fig 2) were performed for both September 2016 and March 2017 samplings. During September period Dinophyceae and Coccolithophores (Haptophyceae) were the dominant groups relative to Bacillariophyceae. All groups showed highest abundances in station F1, whereas the latter group was totally missing from stations P3, P5 and L5. In March 2017, Bacillariophyceae displayed higher values than Dinophyceae; values still indicate an oligotrophic environment. Coccolithophores were relatively higher in respect to September sampling but still represented a minor assemblage component.

During the warm-period samplings (September 2011, 2016; S1 and S2 Appendixs), SEM analyses revealed a total of 73 coccolithophore species out of which 34 were holococcolithophores.

Total coccospheres (Tables 1 and 3) reached up to 25 x 10^3^ cells l^-1^ at P1/5 m in September 2011, whereas maximum values exceeded 39 x 10^3^ cells l^-1^ in P1/2 m during September 2016 (max. mean values 31.74 x 10^3^ cells l^-1^ in P1/0–20 m; Table 3). The maximum cell abundances of the latter sampling have been documented for station F1/2 m (54 x 10^3^ cells l^-1^).

**Table 3:** Coccolithophore community structure. Hetrococcolithophore and holococcolithophore densities in the different sampling periods and the different sampling sites in Methana and Andros Island.

Station	Water depth class (m)	Time period	Mean total coccospheres 10^3^ cells l^-1^	Mean total holococcolithophores 10^3^ cells l^-1^	Mean total holococcolithophore percentage	Mean total heterococcolithophores 10^3^ cells l^-1^	Mean total heterococcolithophore percentage
P1	0–20	Sept-2011	24.41	18.31	75.00	6.1	25
P1	0–20	Sept-2016	31.74	25.06	80.97	6.68	19.03
P1	0–20	March-2017	7.80	0.00	0.00	7.8	100
P2	0–20	Sept-2011	5.01	0.56	11.11	4.45	88.88
P2	0–20	Sept-2016	23.68	9.15	65.78	8.10	34.22
P2	0–20	March-2017	5.47	0.00	0.00	5.47	100
P3	0–20	Sept-2016	26.41	22.26	84.03	4.14	15.97
P3	0–20	March-2017	2.57	0.00	0.00	2.57	100
P3	40–60	Sept-2016	27.40	5.05	14.84	22.35	85.16
P3	40–60	March-2017	2.76	0.00	0.00	2.76	100
P4	0–20	Sept-2016	15.27	8.74	57.84	6.53	42.16
P4	0–20	March-2017	8.72	0.00	0.00	8.72	100
P4	40–60	Sept-2016	24.26	5.50	22.25	18.76	77.75
P4	40–60	March-2017	7.65	0.00	0.00	7.65	100
P5	0–20	Sept-2016	30.96	21.91	70.71	9.05	29.29
P5	0–20	March-2017	4.38	0.49	11.11	3.89	88.89
F1	0–20	Sept-2016	26.79	11.94	64.37	6.59	36.63
F1	0–20	March-2017	9.24	0.00	0.00	9.24	100
R1	0–20	Sept-2016	18.52	11.35	42.35	15.44	57.65
R1	0–20	March-2017	5.80	4.09	70.59	1.71	29.41
L5	0–20	Sept-2016	21.09	13.25	62.86	7.83	37.14
L5	0–20	March-2017	no data	no data	no data	no data	no data
ANDROS-T3-1	0–20	Aug-2001	9.99	2.69	29.33	7.30	70.67
ANDROS-T3-1	0–20	Aug-2002	6.85	2.61	37.67	4.24	62.33
ANDROS-T1-100	0–15	Sept-2004	8.99	3.82	39.56	5.17	60.44

**Fig 3:**
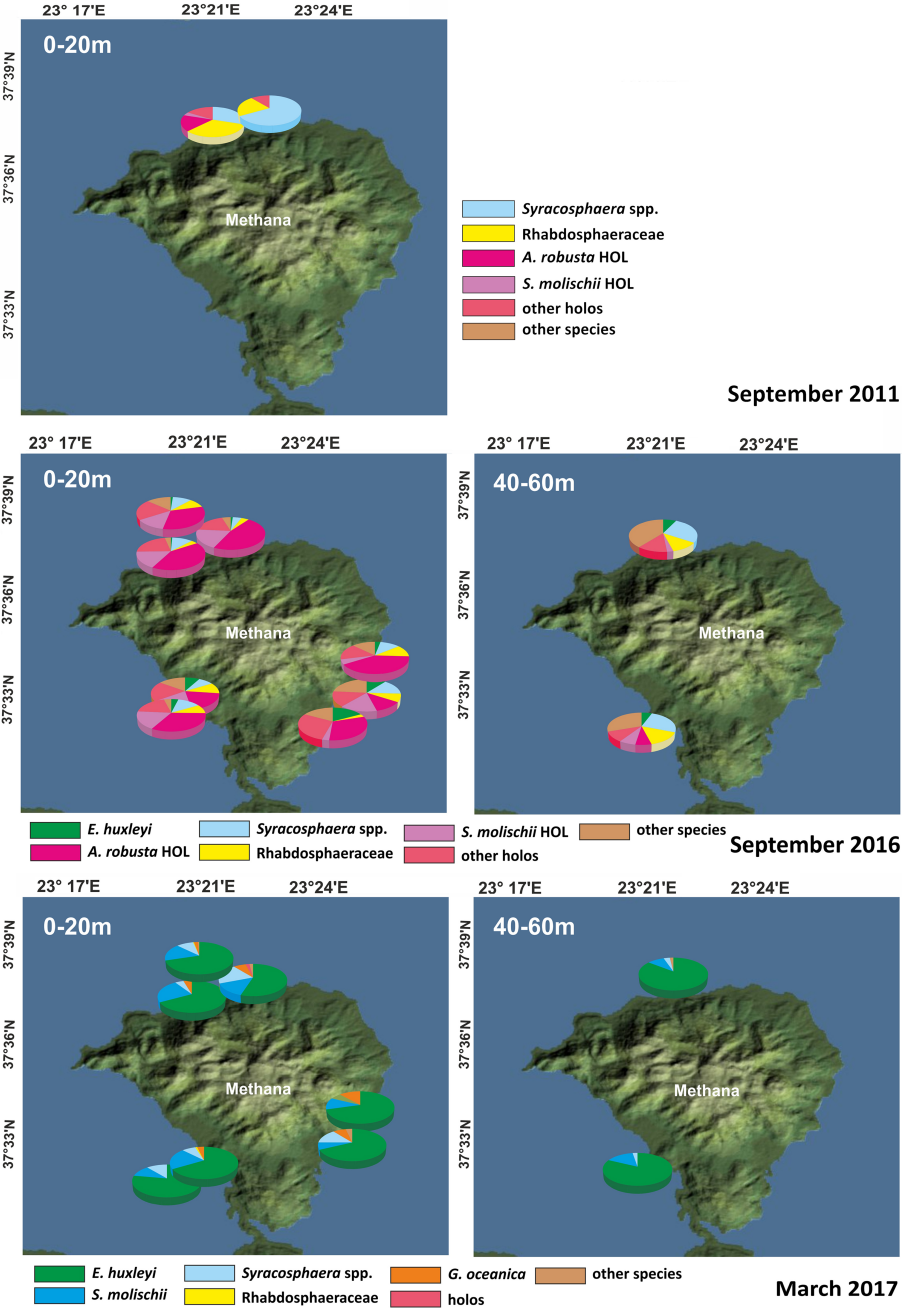
Coccolithophore species composition. Relative abundance of coccolithophore species during September 2011 sampling, September 2016 and March 2017 samplings. Image resource: NASA Worldview.

Concerning species composition, *Syracosphaera* spp. comprised 30–60% of the total coccolithophore assemblage during September 2011, followed by Rhabdosphaeraceae; interestingly *E*. *huxleyi* was totally absent (Fig 3 and S1 Appendix). Holococcolithophores exceeded 18 x 10^3^ cells l^-1^ (>70%) at P1/5 m (Table 3 and S1 Appendix), with *Algirosphaera robusta* HOL (“*Sphaerocalyptra quadridentata”*) being the dominant taxon with up to 8.55 x 10^3^ cells l^-1^, 35% of the coccolithophore assemblage (Fig 3 and S1 Appendix).

During September 2016, stations P1-P3 were characterized by the presence of both hetero- and holococcolithophore species with the latter exhibiting particularly high values (Fig 3and Tables 1 and 3). Numerous different holococcolithophore species (see S2 Appendix) were representing more than 60% of the surface water assemblages in most samples. Water collected close to the main CO_2_ seeps had the highest concentrations of holococcolithophores (max. ~30 x10^3^ cells l^-1^, 90% in relative abundance; P1-5 m). *Algirosphaera robusta* HOL was again dominating the coccolithophore communities exceeding 40–50% in P1/0-20 m and P3/0-20 m (up to 16.5 x 10^3^ cells l^-1^; S1 Appendix). It presented increased values (>30%) in P5/0-20 m and it was also abundant (>40%) in R1/0-20 m (8 x 10^3^ cells l^-1^).

Out of the heterococcolithophores, Syracosphaeraceae and Rhabdosphaeraceae were contributing usually > 10% to the assemblages (S1 Appendix and Table 2). In contrast, *Emiliania huxleyi* displayed very low cell densities during the September 2016 sampling with minimum cell concentrations of 0.6 x 10^3^ cells l^-1^ in P1, P2/0–20 m (<1%), and maximum abundances of ~6 x 10^3^ cells l^-1^ at F1/0–20 m. In March 2017, total coccospheres displayed much lower values (Table 1; max. 10 x 10^3^ cells l^-1^ at P1/0–20 m and F1/0–20 m and max. mean values 9.24 x 10^3^ cells l^-1^ at F1/0–20 m; Table 3).

Species composition was completely different in March 2017 in comparison to September samplings, with *E*. *huxleyi* being dominant with values >60% in all stations. Syracosphaeraceae represented the second most important group, whereas holococcolithophores were practically absent.

*H’* index median values were mostly >1 and > 2 for September 2011 and September 2016 datasets, whereas they were <1 for March 2017 samples (Table 1).

## Discussion

The Methana vent site represents an extended submarine volcanic field area of CO_2_ seeps with observed effects of ocean acidification. Recent data on the macroalgal community of the Methana seep site have shown that benthic communities decreased in calcifying algal cover and increased in brown algal cover with increasing pCO_2_ [37] and skeletal degradation in sea urchin species was observed followed by remarkable increases in skeletal manganese levels [38].

**Fig 4:**
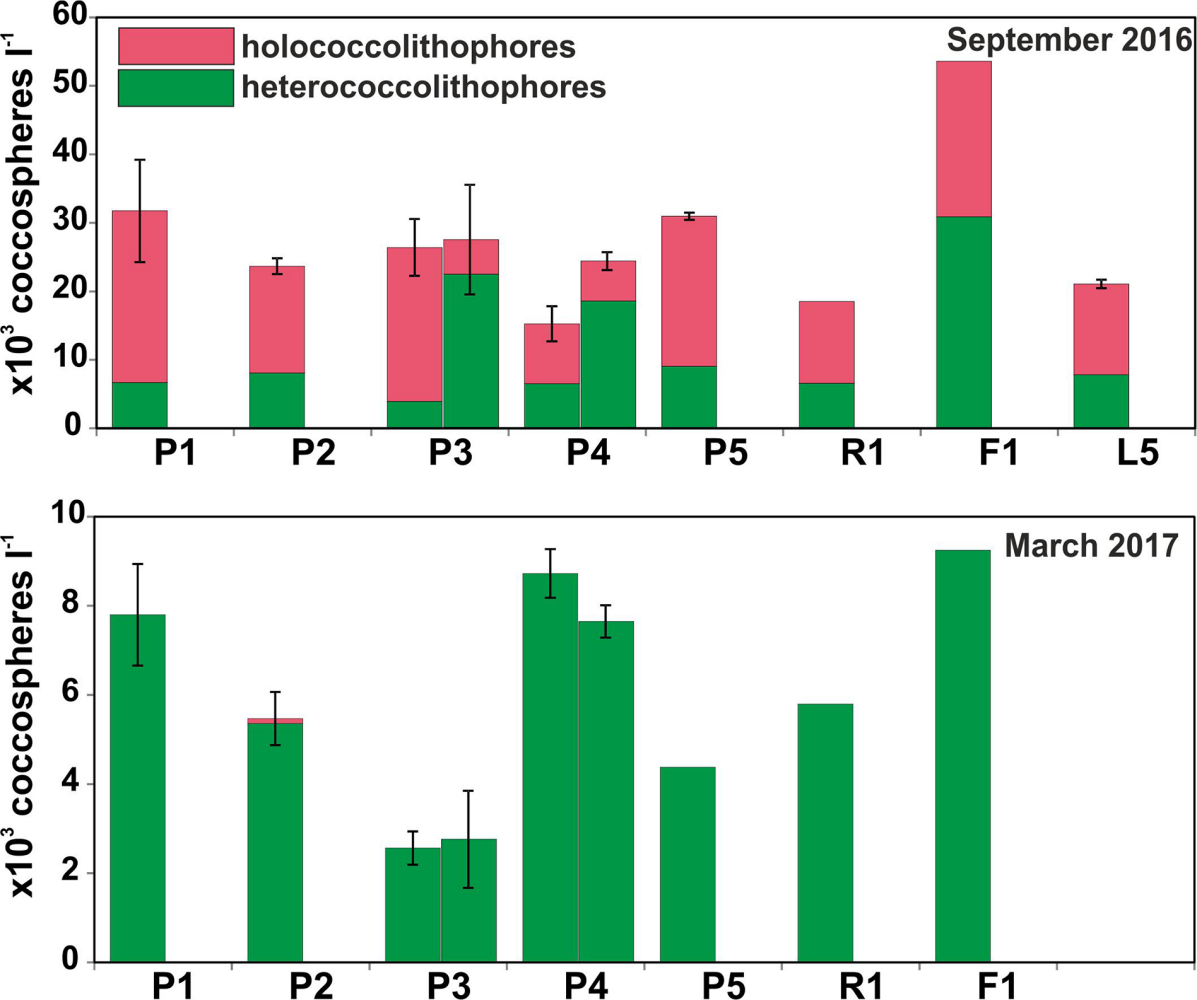
Coccolithophore community structure. Heterococcolithophore-holococcolithophore ratios in the sampling sites during the different sampling periods.

**Fig 5:**
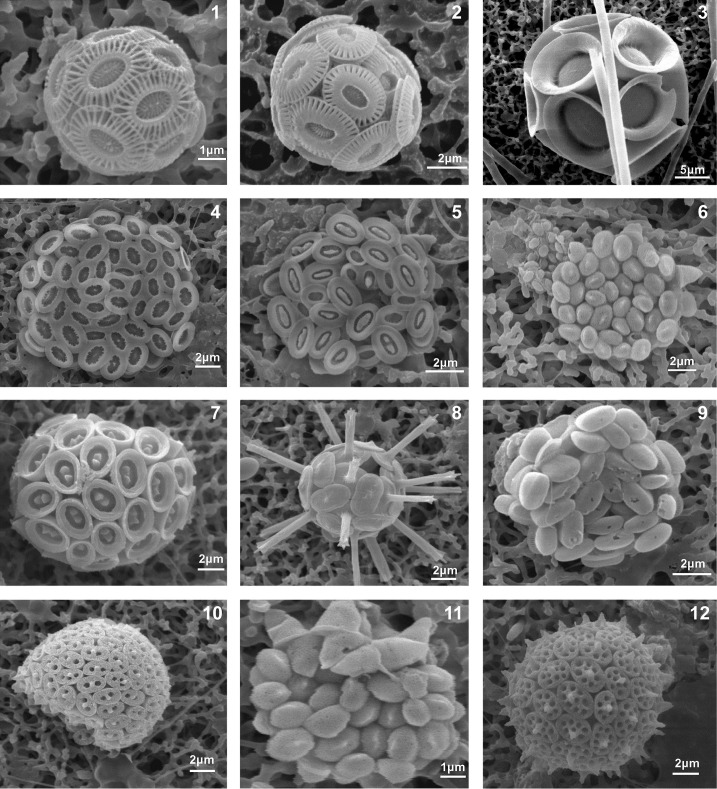
Coccolithophores of Methana acidified environments. 1. *E*. *huxleyi*, P1-5 m, September 2016 (Ωmin<1). 2. *E*. *huxleyi*, P2-5 m, March 2017 (pH<8). 3. *Pontosphaera syracusana*, P2-2 m, March 2017. 4. *Syracosphaera halldalii*, P1-2 m, September 2016 (Ωmin<1). 5. *Syracosphaera ossa*, P1-2 m, September 2016. 6. *Algyrosphaera robusta* HOL, P1-2 m, September 2016 (Ωmin<1). 7. *Syracosphaera mediterranea*, P1-2 m, September 2016 Ωmin<1). 8. *Rhabdosphaera clavigera*, P1-2 m, September 2016 (Ωmin<1). 9. *Algyrosphaera robusta*, P1-2 m, September 2016 (Ωmin<1). 10. *Syracolithus ponticuliferus*, P1-2 m, September 2016 (Ωmin<1). 11. *Algyrosphaera robusta* HOL, P1-2 m, September 2016 (Ωmin<1). 12. *Syracosphaera mediterranea* HOL *wettsteinii* type, P1-2 m, September 2016 (Ωmin<1).

**Fig 6:**
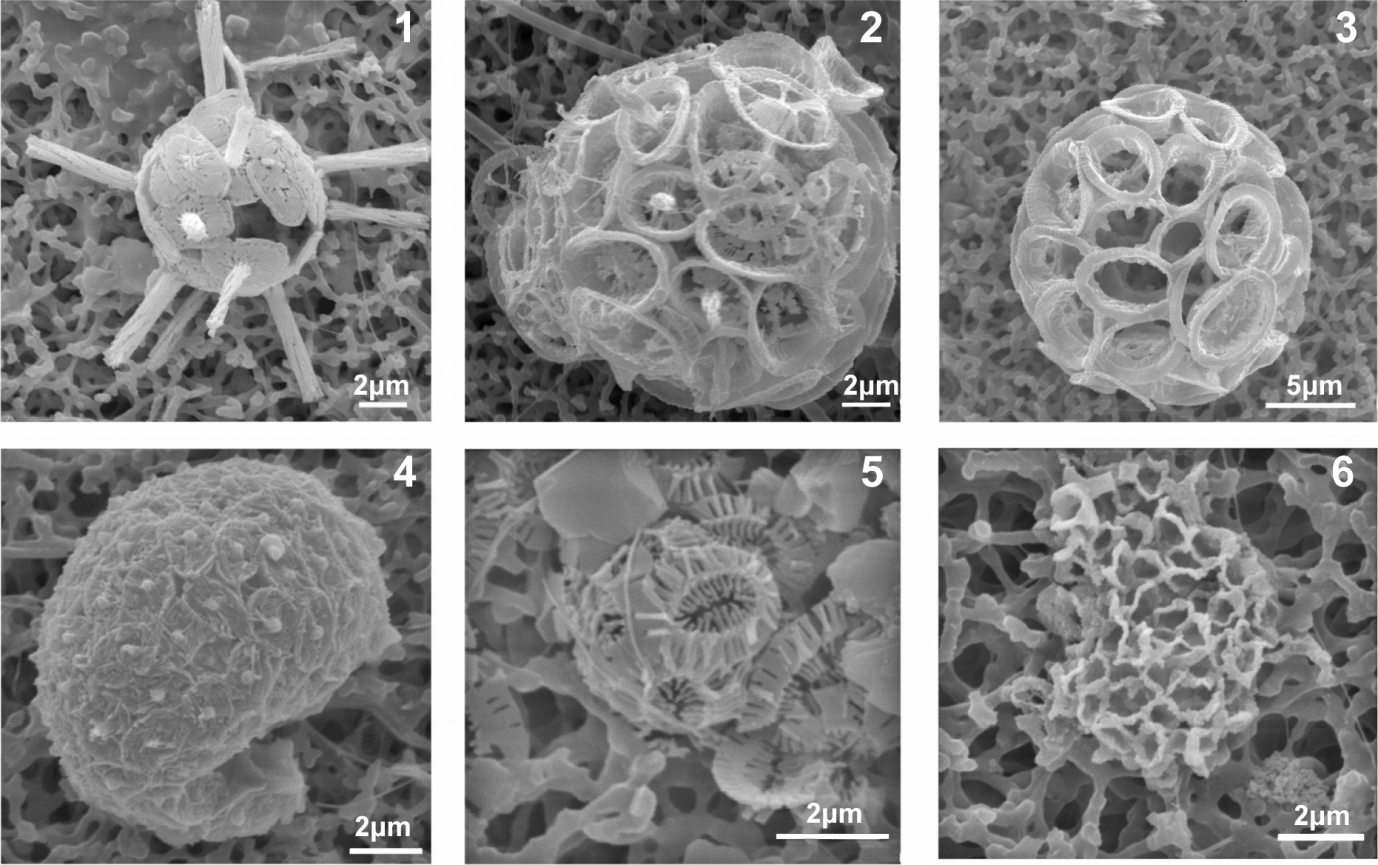
Coccolithophore corroded specimens in Methana acidified environments (Ω<1). 1. *Rhabdosphaera clavigera*, P2-2 m, September 2016. 2. *Syracosphaera pulchra*, P2-8 m, September 2011. 3. *Syracosphaera pulchra*, P2-20 m, September 2011. 4. *Syracosphaera mediterranea* HOL *(hellenica)*, P1-20 m, September 2011. 5. *Emiliania huxleyi*, P1-2 m, September 2016. 6. *Homozygosphaera arethusae*, P1-2 m, September 2016.

**Fig 7:**
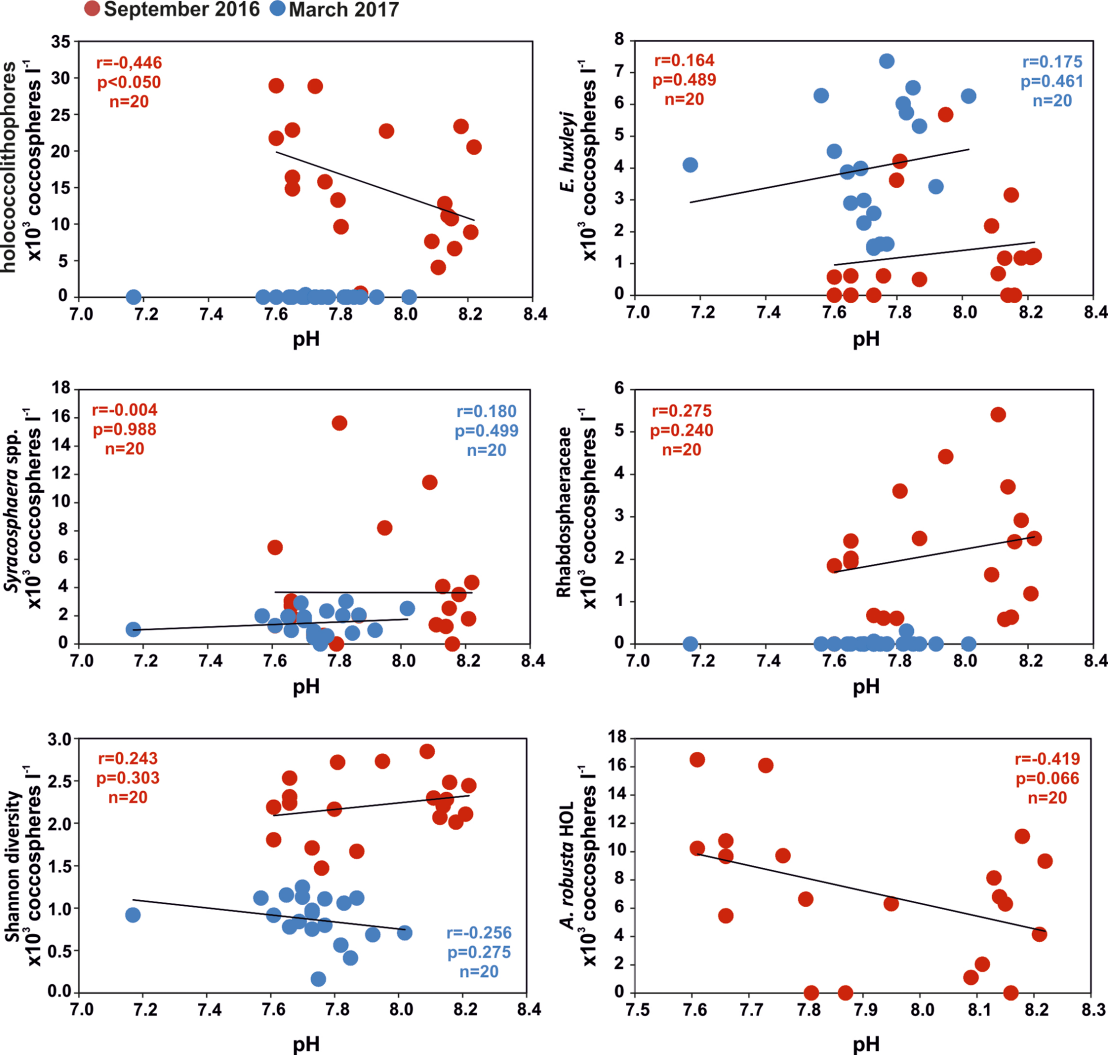
Corellation of various coccolithophore groups and coccolithophore diversity with *in situ* pH data. Holococcolithophores and particularly *A*. *robusta* HOL showed a clear increasing trend with lower pH during the warm period (September 2016), forcing diversity (*H’*) to display an opposite pattern. (obtained p values below 0.05 indicate statistically significant correlation at the 95% confidence level).

Within our coccolithophore study, both September samplings off Methana have taken place in distinctively warm, oligotrophic and stratified waters. Values of pH below 8 vary both spatially and seasonally in an extended area around Methana peninsula; the P1 station that represents a shallow area with documented CO_2_ bubbles seeping from the sea floor (37, this study]) constantly displayed low pH values and Ω<1 (Table 2). Current flows at this part of Saronikos Gulf are very weak during the warm period (10 cm/sec; Fig 1B) and as having a NW direction [47] do not essentially affect the study area at the northwestern part of Methana peninsula, especially during the September samplings. Hence, it is anticipated that coccolithophore assemblages, given that as nannoplanktonic organisms up to a few days or weeks (De Vargas et al., 2004) [65] with mobility functions ranging between 0.1 and 10 m per day (Young, 1994) [66], are practically exposed in the acidified water bodies around the vent area throughout their entire life duration. Surprisingly, despite the low pH values and the undersaturated conditions in both calcite and aragonite, especially at station P1 (Table 2), holococcolithophores are thriving in higher numbers (Fig 4; max. ~30 x10^3^ cells l^-1^) than what has been observed in similar environmental settings with “normal” pH values at the coastal environments off Andros Island, central Aegean Sea, where holococcolithophore total abundance was up to 6.1 x 10^3^ cells l^-1^ in the warm-period samplings (Fig 1A and Table 3) [67, 68]). Our data off Methana indicate that both holococcolithophores (during the warm season; Fig 4) and heterococcolithophores (mostly *E*. *huxleyi*, during the cold months; Fig 4) are unaffected in terms of abundance by low pH environment and presumably undersaturated conditions and also maintain their coccolith structure intact (Fig 5). Corroded coccospheres of both hetero- and holococcolithophore specimens have been found as very rare (<1%; Fig 6). The striking difference in community structure between September and March represents the seasonal variability of the Aegean Sea with *E*. *huxleyi* and Syracosphaeraceae prevailing in the high cell density and low diversity assemblages during the winter and early spring, under low temperatures and higher nutrient concentrations [45 ] (Tables 1 and 2, data in S1 Appendix). Interestingly holococcolithophores and especially *A*. *robusta* HOL display increasing trend with lower pH, whereas diversity is showing a weak decreasing trend apparently associated with the dominance of *A*. *robusta* HOL (Fig 7). Our findings are thus in contrast to coccolithophore field data from the Vulcano vent site [25], which revealed a progressive decrease in coccolithophore diversity and cell concentration with decreasing pH and Ω _calc_ values. Furthermore, in that study corroded and malformed specimens of *E*. *huxleyi* were observed near the seeps (pH 6.84, Ω<1); nevertheless, the authors have also reported holococcolithophores to be found at the lowest pH stations.

The notably high abundance of holococcolithophores, that are known to form high-Mg coccoliths, as extracellular coccolithophore calcifiers [69, 70] at Methana site is quite unexpected as ocean waters with an Ω <1.0 normally lead to carbonate dissolution [e.g. 17, 71, 72, 73]. Whitman Miller et al. [74] have stated that as the saturation state reduces, biomineralisation is expected to become more energetically expensive. Indeed, Gibbs et al. [75] have used the distribution of the extracellular calcifying holococcoliths across the Paleocene-Eocene Thermal Maximum, as a novel indicator of biomineralization in order to assess ocean acidification response. Although extracellular calcification may be more sensitive to changes in seawater chemistry, Gibbs et al. [75] showed that the effects of ocean acidification were only evidenced when paired with elevated temperatures, in accordance with the outcome of previous studies [e.g. 11, 76, 77,78]. Interestingly, similar findings were observed during a mesocosm experiment performed at the CRETACOSMOS mesocosm facility in HCMR Crete, where acidification alone (amendment to IPCC 2100 predictions) seemed to produce a short term enhancement of total phytoplankton biomass; warming alone had a similar effect but on primary production while acidification coupled to warming (greenhouse effect) seemed to further enhance the observed responses of phytoplankton community to each climatic stressor, respectively [79]. In addition, an earlier study of Feely et al. [80] already suggested that the response of marine calcifiers to decreasing calcium carbonate saturation state will be species-specific, depending on environmental parameters such as light, temperature and available nutrients, carbonate mineralogy and calcification mechanisms. In the haptophytes, heteromorphic life cycles with alternation of haploid and diploid stages produced via meiosis and syngamy are widespread or even ubiquitous [81, 82]. At present, limited numbers of complete life cycles are known in extant coccolithophores [67, 82–87]. However, strong evidence suggests that the ecological preferences of the haploid and diploid generation are distinct. The K-selected group of holococcolithophores is more common and diverse under increased light conditions in the surface layers of oligotrophic, warm and stratified environments [45, 68,88–89] and increases in abundance towards shallower depths [e.g., 67].

Recent field data from a Mediterranean transect [90] verified the ability of coccolithophore haplo-diploid life cycle to adapt to the relatively high calcite saturation state, high temperature, stratified and oligotrophic south-eastern Mediterranean waters. Methana field data from the present study provide evidence of spectacular resistance of holococcolithophores in decreased pH conditions (Figs 5 and 6).

Noel et al. [91] already suggested the importance of seawater temperature and chemical composition in coccolithophore life-cycle transitions, however thriving of high-Mg holococcolithophores in low-saturated waters of a dinoflagellate dominated-world (Fig 2), needs further explanation, especially when a certain holococcolithophore species, *A*. *robusta* HOL with distinctively high Mg values [70], is prevailing (Fig 5). As temperature has proven to play the crucial role to potential acidification impacts [75], it appears that our field data, although preliminary, document negligible acidification effects in oligotrophic to ultra-oligotrophic waters and temperatures below 28° C. Apparently a simple temperature threshold does not adequately explain holococcolithophore distribution and the low nutrient availability in relation to saturation variability should be taken into account; however this remains to be further tested by advanced environmental monitoring and laboratory bioassay experiments.

## Conclusions

Assemblages of living coccolithophores were investigated off Methana, eastern Peloponnese peninsula (Greece), along a pH gradient formed by natural CO_2_ seeps. High numbers of holococcolithophore species were dominating the assemblages in the surface water during September. Assemblages were unaffected by low pH environment and undersaturated conditions; surprisingly, holococcolithophores and in particular *Algirosphaera robusta* HOL displayed an increasing trend with lower pH. *Emiliania huxleyi* was present only in low relative abundances in September samples, whereas it was more common in March. However, no malformed and very few corroded coccoliths were observed. Changes in the community structure should rather be related to increased temperatures and nutrient content, while the overall trend associates low pH values with high cell densities. Only diversity showed a weak decreasing trend, apparently associated with the dominance of *A*. *robusta* HOL.

## Supporting information

S1 Appendix Coccolithophore absolute abundances (10 ^3^ cells l^-1^) and relative abundances (%) at the investigated samples. (XLSX)

S2 Appendix Coccolithophore species identified in this study. (XLSX)

## References

[1] Climate Change 2014: Impacts, Adaptation, and Vulnerability. Part A: Global and Sectoral Aspects. Contribution of Working Group II to the Fifth Assessment Report of the Intergovernmental Panel on Climate Change. In: Field C B, Barros V R., Dokken D J, et al, editors. IPCC. 2014. Cambridge, United Kingdom.

[2] Sabine CL, Feely RA, Gruber N, Key RM, Lee K, Bullister JL, et al The Oceanic Sink for Anthropogenic CO_2_. Science. 2004; 305:367–71. doi: 10.1126/science.1097403 15256665 10.1126/science.1097403

[3] Canadell JG, Le Quéré C, Raupach MR, Field CB, Buitenhuis ET, Ciais P, et al Contributions to accelerating atmospheric CO_2_ growth from economic activity, carbon intensity, and efficiency of natural sinks. Proc Nat Acad Sci. 2007; 104(47):18866–70. doi: 10.1073/pnas.0702737104 17962418 10.1073/pnas.0702737104 PMC2141868

[4] Le Quere C. Closing the global budget for CO_2_. Glob Chang. 2009; 74:28–31. doi: 10.1073/pnas.0702737104 17962418

[5] Doney SC, Fabry VJ, Feely RA, Kleypas JA. Ocean Acidification: The Other CO_2_ Problem. Ann Rev Mar Sci. 2009; 1(1):169–92. doi: 10.1146/annurev.marine.010908.163834 21141034 10.1146/annurev.marine.010908.163834

[6] Feely RA, Orr J, Fabry VJ, Kleypas JA, Sabine CL, Langdon C. Present and future changes in seawater chemistry due to ocean acidification. Geophys Monograph Ser. 2009; 183:175–188. doi: 10.1029/2005GM000337

[7] Raven J, Caldeira K, Elderfield H, Hoegh-Guldberg O, Liss PS, Riebesell U, et al Ocean Acidification due to Increasing Atmospheric Carbon Dioxide. Royal Society Policy Document; 2005:1–60.

[8] Caldeira K, Wickett M. Anthropogenic carbon and ocean pH. Nature. 2003; 425: (6956):365 doi: 10.1038/425365a 14508477 10.1038/425365a

[9] Hönisch B, Ridgwell A, Schmidt DN, Thomas E, Gibbs SJ, Sluijs A, et al The Geological Record of Ocean Acidification. Science. 2012; 335:1058–63. doi: 10.1126/science.1208277 22383840 10.1126/science.1208277

[10] Kroeker KJ, Kordas RL, Crim R, Hendriks IE, Ramajo L, Singh GS, et al Impacts of ocean acidification on marine organisms: quantifying sensitivities and interaction with warming. Glob Chang Biol. 2013; 19(6):1884–96. doi: 10.1111/gcb.12179 23505245 10.1111/gcb.12179 PMC3664023

[11] Kroeker KJ, Micheli F, Gambi MC. Ocean acidification causes ecosystem shifts via altered competitive interactions. Nature Clim Change. 2013; 3:156–159. doi: 10.1038/nclimate1680

[12] Kroeker KJ, Kordas RL, Crim RN, Singh GG. Meta-analysis reveals negative yet variable effects of ocean acidification on marine organisms. Ecol Lett. 2010; 13:1419–34. doi: 10.1111/j.1461-0248.2010.01518.x 20958904 10.1111/j.1461-0248.2010.01518.x

[13] Ries JB, Cohen AL, McCorkle DC. Marine calcifiers exhibit mixed responses to CO_2_-induced ocean acidification. Geology. 2009; 37(12):1131–4. https://doi.org/10.1130/G30210A.1.

[14] Orr JC, Fabry VJ, Aumont O, Bopp L, Doney SC, Feely RA, et al Anthropogenic ocean acidification over the twenty-first century and its impact on calcifying organisms. Nature. 2005; 437:681–686. doi: 10.1038/nature04095 16193043 10.1038/nature04095

[15] Riebesell U, Zondervan I, Rost B, Tortell PD, Zeebe RE, Morel FMM. Reduced calcification of marine plankton in response to increased atmospheric CO_2_. Nature. 2000;407:364–367. doi: 10.1038/35030078 11014189 10.1038/35030078

[16] Riebesell U, Schulz KG, Bellerby RGJ, Botros M, Fritsche P, Meyerhöfer M, et al Enhanced biological carbon consumption in a high CO_2_ ocean. Nature. 2007; 450:545–548. doi: 10.1038/nature06267 17994008 10.1038/nature06267

[17] Fabry VJ, Seibel BA, Feely RA, Orr JC. Impacts of ocean acidification on marine fauna and ecosystem processes. ICES J Mar Sci. 2008; 65(3):414–32. doi: 10.1093/icesjms/fsn048

[18] Iglesias-Rodriguez MD, Halloran PR, Rickaby REM, Hall IR, Colmenero-Hidalgo E, Gittins JR, et al Phytoplankton Calcification in a High-CO_2_ World. Science. 2008; 320(5874):336–340. doi: 10.1126/science.1154122 18420926 10.1126/science.1154122

[19] Wood HL, Spicer JI, Widdicombe S. Ocean acidification may increase calcification rates, but at a cost. Proc R Soc B Biol Sci. 2008; 275(1644):1767–73. doi: 10.1098/rspb.2008.0343 18460426 10.1098/rspb.2008.0343 PMC2587798

[20] Ziveri P, Meier KS, Auliaherliaty L, Beaufort L, Stoll HM, Triantaphyllou M, et al Impact of acidification on pelagic calcifying organisms in the Mediterranean Sea. In: CIESM Monogr. 2008; 36: 99–101.

[21] Langer G, Nehrke G, Probert I, Ly J, Ziveri P. Strain-specific responses of *Emiliania huxleyi* to changing seawater carbonate chemistry. Biogeosciences. 2009; 6(11):2637–46. doi: 10.5194/bg-6-2637-2009

[22] Beaufort L, Probert I, Buchet N. Effects of acidification and primary production on coccolith weight: Implications for carbonate transfer from the surface to the deep ocean. Geochem Geophys Geosystems. 2007; 8(8). doi: 10.1029/2006GC001493

[23] Hall-Spencer JM, Rodolfo-Metalpa R, Martin S, Ransome E, Fine M, Turner SM, et al Volcanic carbon dioxide vents show ecosystem effects of ocean acidification. Nature. 2008; 454(7200):96–99. doi: 10.1038/nature07051 18536730 10.1038/nature07051

[24] Wall-Palmer D, Smart CW, Hart MB. In-life pteropod shell dissolution as an indicator of pastocean carbonate saturation. Quat Sci Rev. 2013; 81(Supplement C):29–34. doi: 10.1016/j.quascirev.2013.09.019

[25] Ziveri P, Passaro M, Incarbona A, Milazzo M, Rodolfo-Metalpa R, Hall-Spencer JM. Decline in coccolithophore diversity and impact on coccolith morphogenesis along a natural CO_2_ gradient. Biol Bull. 2014; 226(3):282–90. doi: 10.1086/BBLv226n3p282 25070871 10.1086/BBLv226n3p282

[26] Bethoux JP, Gentili B, Morin P, Nicolas E, Pierre C, Ruiz-Pino D. The Mediterranean Sea: a miniature ocean for climatic and environmental studies and a key for the climatic functioning of the North Atlantic. Progr Oceanogr. 1999; 44(1–3):131–46. doi: 10.1016/S0079-6611(99)00023-3

[27] Pinardi N, Masetti E. Variability of the large scale general circulation of the Mediterranean Sea from observations and modelling: a review. Palaeogeogr Palaeoclimatol Palaeoecol. 2000; 158(3–4):153–73. doi: 10.1016/S0031-0182(00)00048-1

[28] Lionello P, Malanotte-Rizzoli P, Boscolo R, Alpert P, Artale V, Li L, et al The Mediterranean climate: An overview of the main characteristics and issues. Develop Earth Environ Sci. 2006; 4:1–26. doi: 10.1016/S1571-9197(06)80003-0

[29] Turley CM. The changing Mediterranean Sea a sensitive ecosystem? Prog Oceanogr. 1999; 44(1–3):387–400. doi: 10.1016/S0079-6611(99)00033-6

[30] Gambaiani DD, Mayol P, Isaac SJ, Simmonds MP. Potential impacts of climate change and greenhouse gas emissions on Mediterranean marine ecosystems and cetaceans. J Mar Biol Assoc United Kingdom. 2008; 89(1):179–201. doi: 10.1017/S0025315408002476

[31] Chevaldonne P, Lejeusne C. Regional warming-induced species shift in north-west Mediterranean marine caves. Ecol Lett; 2003; 6(4):371–9. doi: 10.1046/j.1461-0248.2003.00439.x

[32] Rodolfo-Metalpa R, Lombardi C, Cocito S, Hall-Spencer JM, Gambi MC. Effects of ocean acidification and high temperatures on the bryozoan *Myriapora truncata* natural CO_2_ vents. Mar Ecol. 2010; 31(3):447–56. doi: 10.1111/j.1439-0485.2009.00354.x

[33] Johnson VR, Russell BD, Fabricius KE, Brownlee C, Hall-Spencer JM. Temperate and tropical brown macroalgae thrive, despite decalcification, along natural CO_2_ gradients. Glob Chang Biol. 2012; 18(9):2792–803. doi: 10.1111/j.1365-2486.2012.02716.x 24501057 10.1111/j.1365-2486.2012.02716.x

[34] Johnson VR, Brownlee C, Rickaby REM, Graziano M, Milazzo M, Hall-Spencer JM. Responses of marine benthic microalgae to elevated CO_2_. Mar Biol. 2011; 160(8):1813–24.

[35] Boatta F, D’Alessandro W, Gagliano AL, Liotta M, Milazzo M, Rodolfo-Metalpa R, et al Geochemical survey of Levante Bay, Vulcano Island (Italy), a natural laboratory for the study of ocean acidification. Mar Pollut Bull. 2013; 73(2):485–94. doi: 10.1016/j.marpolbul.2013.01.029 23465567 10.1016/j.marpolbul.2013.01.029

[36] Milazzo M, Rodolfo-Metalpa R, Chan VBS, Fine M, Alessi C, Thiyagarajan V, et al Ocean acidification impairs vermetid reef recruitment. Sci Rep. 2014; 4(1):4189 doi: 10.1038/srep04189 24577050 10.1038/srep04189 PMC5379440

[37] Baggini C, Salomidi M, Voutsinas E, Bray L, Krasakopoulou E, Hall-Spencer JM. Seasonality Affects Macroalgal Community Response to Increases in pCO_2_. PLoS One. 2014; 9(9):e106520 doi: 10.1371/journal.pone.0106520 25184242 10.1371/journal.pone.0106520 PMC4153631

[38] Bray L, Pancucci-Papadopoulou MA, Hall-Spencer JM. Sea urchin response to rising pCO_2_ shows ocean acidification may fundamentally alter the chemistry of marine skeletons. Med Mar Sci. 2014; 15(3):510 http://doi.org/10.12681/mms.579.

[39] Ziveri P, Rutten A, De Lange G, Thomson J, Corselli C. Present-day coccolith fluxes recorded in central eastern Mediterranean sediment traps and surface sediments. Palaeogeogr Palaeoclimatol Palaeoecol. 2000;158:175–195.

[40] Triantaphyllou M V, Ziveri P, Tselepides A. Coccolithophore export production and response to seasonal surface water variability in the oligotrophic Cretan Sea (NE Mediterranean). Micropaleontology. 2004; 50(Suppl1):127–44. doi: 10.2113/50.Suppl_1.127

[41] Malinverno E, Maffioli P, Corselli C, De Lange G. Present-day fluxes of coccolithophores and diatoms in the pelagic Ionian Sea. J Mar Syst. 2014; 132:13–27.

[42] Schneider A, Wallace DWR, Körtzinger A. Alkalinity of the Mediterranean Sea. Geophys Res Lett. 2007; 34(15). doi: 10.1029/2006GL028842

[43] Parke M, Adams I. The motile (*Crystallolithus hyalinus* Gaarder and Markali) and non-motile phases in the life history of *Coccolithus pelagicus* Schiller. J Mar Biol Assoc UK. 1960; 39: 263–274.

[44] Houdan A, Billard C, Marie D, Not F, Saez AG, Young JR, Probert I. Flow cytometric analysis of relative ploidy levels in holococcolithophore-heterococcolithophore (Haptophyta) life cycles. Syst Biodivers. 2004; 1:453–465.

[45] Dimiza M, Triantaphyllou MV, Malinverno E, Psarra S, Karatsolis BT, Mara P, et al The composition and distribution of living coccolithophores in the Aegean Sea (NE Mediterranean). Micropaleontology. 2015; 61(6):521–40.

[46] Triantaphyllou M, Dimiza M, Krasakopoulou E, Malinverno E, Lianou V, Souvermezoglou E. Seasonal variation in *Emiliania huxleyi* coccolith morphology and calcification in the Aegean Sea (Eastern Mediterranean). Geobios. 2010; 43(1):99–110. doi: 10.1016/j.geobios.2009.09.002

[47] Kontoyiannis H. Observations on the circulation of the Saronikos Gulf: A Mediterranean embayment sea border of Athens, Greece. J Geophys Res. 2010; 115(C6). doi: 10.1029/2008JC005026

[48] Papanikolaou ID, Papanikolaou DI. Seismic hazard scenarios from the longest geologically constrained active fault of the Aegean. Quat Internat. 2007; 171–172:31–44.

[49] Pavlakis P, Papanikolaou D, Chronis G, Lykousis V, Anagnostou C. Geological Structure of Inner Messiniakos Gulf. Bull Geol Soc Greece. 1989; 23: 333–347.

[50] Dando PR, Aliani S, Arab H, Bianchi CN, Brehmer M, Cocito S, et al Hydrothermal studies in the Aegean Sea. Phys Chem Earth, Part B. Hydrol Ocean Atm. 2000; 25(1):1–8. doi: 10.1016/S1464-1909(99)00112-4

[51] Strickland JDH, Parsons TR. A Practical Handbook of Seawater Analysis. Ottawa: Fisheries Res. 1972; 310 p. doi: 10.1002/iroh.19700550118

[52] Rimmelin P, Moutin T. Re-examination of the MAGIC method to determine low orthophosphate concentration in seawater. Anal Chim Acta. 2005; 548(1–2):174–82. doi: 10.1016/j.aca.2005.05.071

[53] Holm-Hansen O, Lorenzen CJ, Holmes RW, Strickland JDH. Fluorometric Determination of Chlorophyll. ICES J Mar Sci. 1965; 30(1):3–15. doi: 10.1093/icesjms/30.1.3

[54] Pierrot D E, Lewis E, Wallace DWR. MS Excel Program Developed for CO2 System Calculations. ORNL/CDIAC-105a. Carbon Dioxide Information Analysis Center, Oak Ridge National Laboratory, U.S. Department of Energy, Oak Ridge, Tennessee. 2006. http://doi.org/10.3334/CDIAC/otg.CO2SYS_XLS_CDIAC105a.

[55] Goyet C, Poisson A. New determination of carbonic-acid dissociation-constants in seawater as a function of temperature and salinity. Deep Sea Res. Part A. Oceanogr Res Pap. 1989; 36 (11): 1635–1654.

[56] Pérez FF, Fraga F. Association constant of fluoride and hydrogen ions in seawater. Mar Chem. 1987; 21: 161–168.

[57] Dickson AG. Standard potential of the reaction: AgCl(s) +1/2H_2_ = Ag(s)+HCl(aq), and the standard acidity constant of the ion HSO4^-^ in synthetic sea water from 273.15 to 318.15 K. J. Chem Thermodyn. 1990; 22: 113–127.

[58] Lee K, Tae-Wook K, Byrne RH, Millero F J, Feely RA, Liu Y-M. The universal ratio of the boron to chlorinity for the North Pacific and North Atlantic oceans. Geochim Cosmochim Acta. 2010; 74: 1801–1811.

[59] Utermöhl H. Zur Vervollkommnung der quantitativen Phytoplankton-Methodik. SIL Commun 1953–1996. 1958; 9(1):1–38.

[60] Sandgren CD, Robinson J V. A stratified sampling approach to compensating for non-random sedimentation of phytoplankton cells in inverted microscope settling chambers. Br Phycol J. 1984; 19(1):67–72. doi: 10.1080/00071618400650071

[61] Jordan RW, Winter A. Living microplankton assemblages off the coast of Puerto Rico during January-May 1995. Mar Micropaleontol. 2000; 39:113–130. doi: 10.1016/S0377-8398(00)00017-7

[62] Young JR, Geisen M, Cros L, Kleune A, Sprengel C, Probert I, et al A guide to extant coccolithophores taxonomy. J Nannoplankton Res. 2003; Special Issue (1):1–25.

[63] Malinverno E, Triantaphyllou M V, Stavrakakis S, Ziveri P, Lykousis V. Seasonal and spatial variability of coccolithophore export production at the South-Western margin of Crete (Eastern Mediterranean). Mar Micropaleontol. 2009; 71(3–4):131–47. doi: 10.1016/j.marmicro.2009.02.002

[64] Hammer Ø, Harper DAT, Ryan PD. PAST: Paleontological Statistics Software Package for Education and Data Analysis. Palaeontol Electronica 2001; 4(1): 9pp.

[65] De Vargas C, Saez AG, Medlin LK, Thierstein HR. Super-Species in the calcareous plankton In: Thierstein HR, Young JR, editors. Coccolithophores. From molecular processes to global impact. Springer 2004; pp. 271–298.

[66] Young JR. Functions of coccoliths In: Winter A, Siesser WG, editors. Coccolithophores. Cambridge University Press 1994; pp. 63–82.

[67] Triantaphyllou MV, Dermitzakis MD, Dimiza MD. Holo- and heterococcolithophorids (calcareous nannoplankton) in the gulf of Korthi (Andros Island, Aegean Sea, Greece) during late summer 2001. Rev Paleobiol. 2002; 21(1):353–69.

[68] Dimiza MD, Triantaphyllou MV, Dermitzakis MD. Seasonality and ecology of living coccolithophores in E. Mediterranean coastal environments (Andros Island, Middle Aegean Sea). Micropaleontology. 2008; 54:159–75.

[69] Rowson JD, Leadbeater BSC, Green JC. Calcium carbonate deposition in the motile (*Crystallolithus*) phase of *Coccolithus pelagicus* (Prymnesiophyceae). Br Phycol J. 1986; 21:359–370.

[70] Cros L, Fortuño J-M, Estrada M. Elemental composition of coccoliths: Mg/Ca relationships. Sci Marina. 2013; 77S1:63–67 doi: 10.3989/scimar.03727.27E

[71] Tyrrell T. Calcium carbonate cycling in future oceans and its influence on future climates. J Plankton Res. 2008; 30(2):141–56. doi: 10.1093/plankt/fbm105

[72] Rodolfo-Metalpa R, Houlbrèque F, Tambutté É, Boisson F, Baggini C, Patti FP, et al Coral and mollusc resistance to ocean acidification adversely affected by warming. Nat Clim Chang. 2011; 1:308 doi: 10.1038/nclimate1200

[73] Jackson EL, Davies AJ, Howell KL, Kershaw PJ, Hall-Spencer JM. Future-proofing marine protected area networks for cold water coral reefs. ICES J Mar Sci. 2014;71(9):2621–9. doi: 10.1093/icesjms/fsu099

[74] Miller AW, Reynolds AC, Sobrino C, Riedel GF. Shellfish Face Uncertain Future in High CO_2_ World: Influence of Acidification on Oyster Larvae Calcification and Growth in Estuaries. PLoS One. 2009; 4(5):e5661 doi: 10.1371/journal.pone.0005661 19478855 10.1371/journal.pone.0005661 PMC2682561

[75] Gibbs SJ, Bown PR, Ridgwell A, Young JR, Poulton AJ, O’Dea SA. Ocean warming, not acidification, controlled coccolithophore response during past greenhouse climate change. Geology. 2015; 44(1):59–6. doi: 10.1130/G37273

[76] Anthony KRN, Maynard JA, Diaz-Pulido G, Mumby PJ, Marshall PA, Cao L, et al Ocean acidification and warming will lower coral reef resilience. Glob Chang Biol. 2011; 17(5):1798–808. doi: 10.1111/j.1365-2486.2010.02364.x

[77] Harvey BP, Gwynn-Jones D, Moore PJ. Meta-analysis reveals complex marine biological responses to the interactive effects of ocean acidification and warming. Ecol Evol. 2013; 3(4):1016–30. doi: 10.1002/ece3.516 23610641 10.1002/ece3.516 PMC3631411

[78] Sinutok S, Hill R, Kühl M, Doblin MA, Ralph PJ. Ocean acidification and warming alter photosynthesis and calcification of the symbiont-bearing foraminifera *Marginopora vertebralis*. Mar Biol. 2014; 161(9):2143–54. doi: 10.1007/s00227-014-2494-7

[79] Tsakalakis I, Lagaria A, Papageorgiou N, Psarra S. Response to warming and acidification of planktonic primary phytoplankton productivity in the oligotrophic Eastern Mediterranean Sea: A mesocosm experiment. 17th Workshop of the International Association of Phytoplankton Taxonomy and Ecology (IAP). 2014.

[80] Feely RA, Sabine CL, Lee K, Berelson W, Kleypas J, Fabry VJ, et al Impact of anthropogenic CO_2_ on the CaCO_3_ system in the oceans. Science. 2004; 305(5682):362–6. doi: 10.1126/science.1097329 15256664 10.1126/science.1097329

[81] Billard C. Life cycles In: Green JC, Leadbeater BSC, editors. The Haptophyte Algae. Clarendon Press, Oxford: The Systematics Association. 1994; Special Volume (51). pp. 167–186.

[82] Houdan A, Billard C, Marie D, Not F, Sáez AG, Young JR, et al Holococcolithophore-heterococcolithophore (Haptophyta) life cycles: Flow cytometric analysis of relative ploidy levels. Syst Biodivers. 2004; 1(4):453–65. doi: 10.1017/S1477200003001270

[83] Cros L, Fortuño JM. Atlas of Northwestern Mediterranean Coccolithophores. Sci Mar. 2002; 66(S1):1–182. doi: 10.3989/scimar.2002.66s11

[84] Geisen M, Billard C, Broerse A, Cros L, Probert I, Young J. Life-cycle associations involving pairs of holococcolithophorid species: intraspecific variation or cryptic speciation? Eur J Phycol. 2002; 37(4):531–50. doi: 10.1017/S0967026202003852

[85] Triantaphyllou MV, Dimiza MD. Verification of the *Algirosphaera robusta*-*Sphaerocalyptra quadridentata* (coccolithophores) life-cycle association. J Micropalaeontol. 2003; 22(1):107–11. doi: 10.1144/jm.22.1.107

[86] Frada M, Percopo I, Young J, Zingone A, de Vargas C, Probert I. First observations of heterococcolithophore-holococcolithophore life cycle combinations in the family Pontosphaeraceae (Calcihaptophycideae, Haptophyta). Mar Micropaleontol. 2009; 71(1–2):20–7. doi: 10.1016/j.marmicro.2009.01.001

[87] Triantaphyllou M, Karatsolis BT, Dimiza M, Malinverno E, Cerino F, Psarra S, et al Coccolithophore combination coccospheres from the NE Mediterranean Sea: new evidence and taxonomic revisions. Micropaleontology. 2015; 61(6):457–72.

[88] Kleijne A. Holococcolithophorids from the Indian Ocean, Red Sea, Mediterranean Sea and North Atlantic Ocean. Mar Micropaleontol. 1991; 17(1–2):1–76.

[89] Cros L, Estrada M. Holo-heterococcolithophore life cycles: ecological implications. Mar Ecol Progr Ser. 2013; 492:57–68. doi: 10.3354/meps10473

[90] D’Amario B, Ziveri P, Grelaud M, Oviedo A, Kralj M. Coccolithophore haploid and diploid distribution patterns in the Mediterranean Sea: can a haplo-diploid life cycle be advantageous under climate change? J Plankton Res. 2017; 39(5):781–94. doi: 10.1093/plankt/fbx044

[91] Nöel MH, Kawachi M, Inouye I. Induced dimorphic life cycle of a coccolithophorid, *Calyptrosphaera sphaeroidea* (Prymnesiophyceae, Haptophyta). J Phycol. 2004; 40(1):112–29. doi: 10.1046/j.1529-8817.2004.03053.x

